# Supplementary material for: Content-rich biological network constructed by mining PubMed abstracts
Source: BMC Bioinformatics. 2004 Oct 8;5:147. doi: 10.1186/1471-2105-5-147 (PMC528731; doi:10.1186/1471-2105-5-147)
Supplement: Additional File 5 — The original Chilibot query results of the term "long-term potentiation (LTP)" and 22 other terms, limiting the latest references analyzed to the years 1990, 1995, 2000, and 2004. [file 1471-2105-5-147-S5.bz2 › chilibotAdditionalFile5/ltp2004/html/PKA_ACTIN.html]

 


 **PKA** and **ACTIN** 
  
Found 119 abstracts in PubMed,  **30 abstracts were retrieved and analyzed**.  


---

 Search Google  |
 PDF files only 
|  EDU domain only 

---

**Interactive relationship** (e.g. stimulation, inhibition, etc)

- Inhibition of  **actin**  polymerization disrupted AKAP79 targeting of  **PKA**  and CaN to ruffles in COS7 cells and endogenous AKAP79 150 dendritic spine localization with  **PKA** , CaN, and PSD MAGUKs in neurons.  Ref: 12177200 J Neurosci, 2002
- Phosphorylation of recombinant lasp 1 with recombinant  **PKA**  increased the K and decreased the B for lasp 1 binding to F  **actin** .  Ref: 12432067 J Cell Sci, 2002
- The results indicate that cyclooxygenase and  **PKA**  stimulate the formation of  **actin**  bundles that contain myosin II and associate with small focal adhesions.  Ref: 12845600 Cell Motil Cytoskeleton, 2003
- Vasodilator stimulated phosphoprotein VASP, an  **actin**  binding protein localized to areas of focal contacts, is a substrate for the cyclic adenosine monophosphate cyclic guanosine monophosphate cAMP cGMP dependent protein kinases  **PKA** , PKG.  Ref: 14706852 FEBS Lett, 2004
- Phosphorylation of spinophilin by  **PKA**  modulated the association between spinophilin and the  **actin**  cytoskeleton.  Ref: 12417592 J Biol Chem, 2003
- Together, these results provide the first evidence that  **PKA**  localization is required for morphogenesis of  **actin**  structures in an intact organism.  Ref: 12223401 Development, 2002
- This localization requires the  **actin**  cytoskeleton but is independent of the regulator CRAC and the effector  **PKA** .  Ref: 12600317 Cell, 2003
- In wild type ovaries,  **Pka**  RII and Akap200 localized to membranes and to the outer rim of ring canals,  **actin**  rich structures that connect germline cells.  Ref: 12223401 Development, 2002

**Parallel relationship** (e.g. studied together, co-existance, homology, etc.)

- Pretreatment with adenosine cyclic monophosphothioate Rp diastereomer Rp cAMPS, a  **PKA**  inhibitor, suppressed the calcitonin induced inhibition of  **actin**  ring formation.  Ref: 14691681 J Bone Miner Metab, 2004
- In contrast, a brief exposure to hypotonic stress decreased the total cellular F  **actin**  content in eel intestinal epithelium by about 15%, detectable morphologically mainly as a decrease in the intensity of the apical brush border F  **actin**  labeling.The bumetanide sensitive response of V te and Isc to hypertonicity was potently inhibited by treatment with either cytochalasin, latrunculin A, colchicine, the protein kinase C PKC inhibitor chelerythrine, the myosin light chain kinase MLCK inhibitor ML 7, or the serine threonine protein phosphatase inhibitor Calyculin A, but was unaffected by the  **PKA**  inhibitor H 89.  Ref: 12297722 Cell Physiol Biochem, 2002
- Vasodilator stimulated phosphoprotein VASP is a regulator of  **actin**  dynamics in platelets and a common substrate of both cAMP and cGMP dependent protein kinases  **PKA**  and PKG.  Ref: 14602552 Platelets, 2003
- Filopodia formation induced by GalNAc S may have a physiological relevance because long term exposure to GalNAc S enhanced F  **actin**  rich dendrite generation of primary cultured hippocampal neurons, and  **PKA**  dependent dendritic outgrowth and branch formation of primary cultured cerebellar Purkinje neurons, in which  **actin**  isoforms were localized to motile structures in dendrites.  Ref: 14643765 Neuroscience, 2003
- We examine the functional importance of  **PKA**  anchoring during Drosophila oogenesis by analyzing membrane integrity and  **actin**  structures in mutants with disruptions in Akap200, an AKAP.  Ref: 12223401 Development, 2002
